# Structure of cytochrome *b*_5_ unique to tardigrades

**DOI:** 10.1002/pro.3896

**Published:** 2020-07-24

**Authors:** Yohta Fukuda, JeeEun Kim, Tsuyoshi Inoue

**Affiliations:** ^1^ Graduate School of Pharmaceutical Science Osaka University Osaka Japan

**Keywords:** cytochrome b5, heme, tardigrade, X‐ray crystallography

## Abstract

Cytochrome *b*_5_ is an essential electron transfer protein, which is ubiquitously found in living systems and involved in wide variety of biological processes. Tardigrades (also known as water bears), some of which are famous for desiccation resistance, have many proteins unique to them. Here, we report spectroscopic and structural characterization of a cytochrome *b*_5_ like protein from one of the desiccation‐tolerant tardigrades, *Ramazzottius varieornatus* strain YOKOZUNA‐1 (*Rv*Cyt*b*_5_). A 1.4 Å resolution crystal structure revealed that *Rv*Cyt*b*_5_ is a new cytochrome *b*_5_ protein specific to tardigrades.

## Abstract 2

PDB Code(s): 7BWH;

## 1 INTRODUCTION

Tardigrades are microscopic multicellular organisms found in almost all environments on Earth from roadside mosses to high mountains and deep sea.^1^ Some terrestrial tardigrades can retain their lives under extremely desiccated conditions through transition into a state called anhydrobiosis, in which metabolic processes are undetectable.^2^, ^3^ In preparation for anhydrobiosis, the bodies of tardigrades gradually shrink to form a “tun.” The tun also shows tolerances to high (151°C)^4^ or low (−273°C)^5^ temperature, exposure to high energy radiations,^6^, ^7^, ^8^ vacuum,^9^, ^10^ high pressure,^11^, ^12^ and toxic chemicals.^13^, ^14^ Moreover, anhydrobiotic tardigrades are known to survive even in space (low Earth orbit) for 10 days.^15^ This extraordinary ability of tardigrades is so famous even in the public that tardigrades appeared in an American famous sci‐fi television series Star Trek and a Japanese magical girl animation series Pretty Cure. On the other hand, the detailed molecular basis of tardigrade anhydrobiosis has been largely ambiguous because their key components, tardigrade‐specific proteins, are poorly investigated at molecular and atomic levels. To understand structure–function relationships of tardigrade‐specific proteins, we have started studies on them with structural biology approaches.^16^, ^17^, ^18^ In this study, we focus on an electron transfer protein, cytochrome *b*_5_ (Cyt*b*_5_), from tardigrades. Electron transfer (ET) plays essential roles in biological processes including photosynthesis and respiration. Cyt*b*_5_ mediates many ET reactions relevant to lipid metabolism,^19^ steroid synthesis,^20^ and methemoglobin reduction.^21^ Because Cyt*b*_5_ is involved in fundamental biological reactions, it is ubiquitously found in animals, plants, and fungi. Besides, amino acid sequences of Cyt*b*_5_ proteins are usually well‐conserved. The genome of one of the toughest tardigrades, *Ramazzottius varieornatus* strain YOKOZUNA‐1, has several structural genes of Cyt*b*_5_ proteins.^22^ Judging from their amino acid sequences, most of them closely resemble well‐known Cyt*b*_5_ proteins; however, one cytochrome *b*_5_ like (Cyt*b*_5_‐like) protein shows low amino acid sequence similarity to them. Here, we present structural and spectroscopic characterization of this unique Cyt*b*_5_‐like protein from *R. varieornatus*.

## 2 RESULTS AND DISCUSSION

**FIGURE 1:**
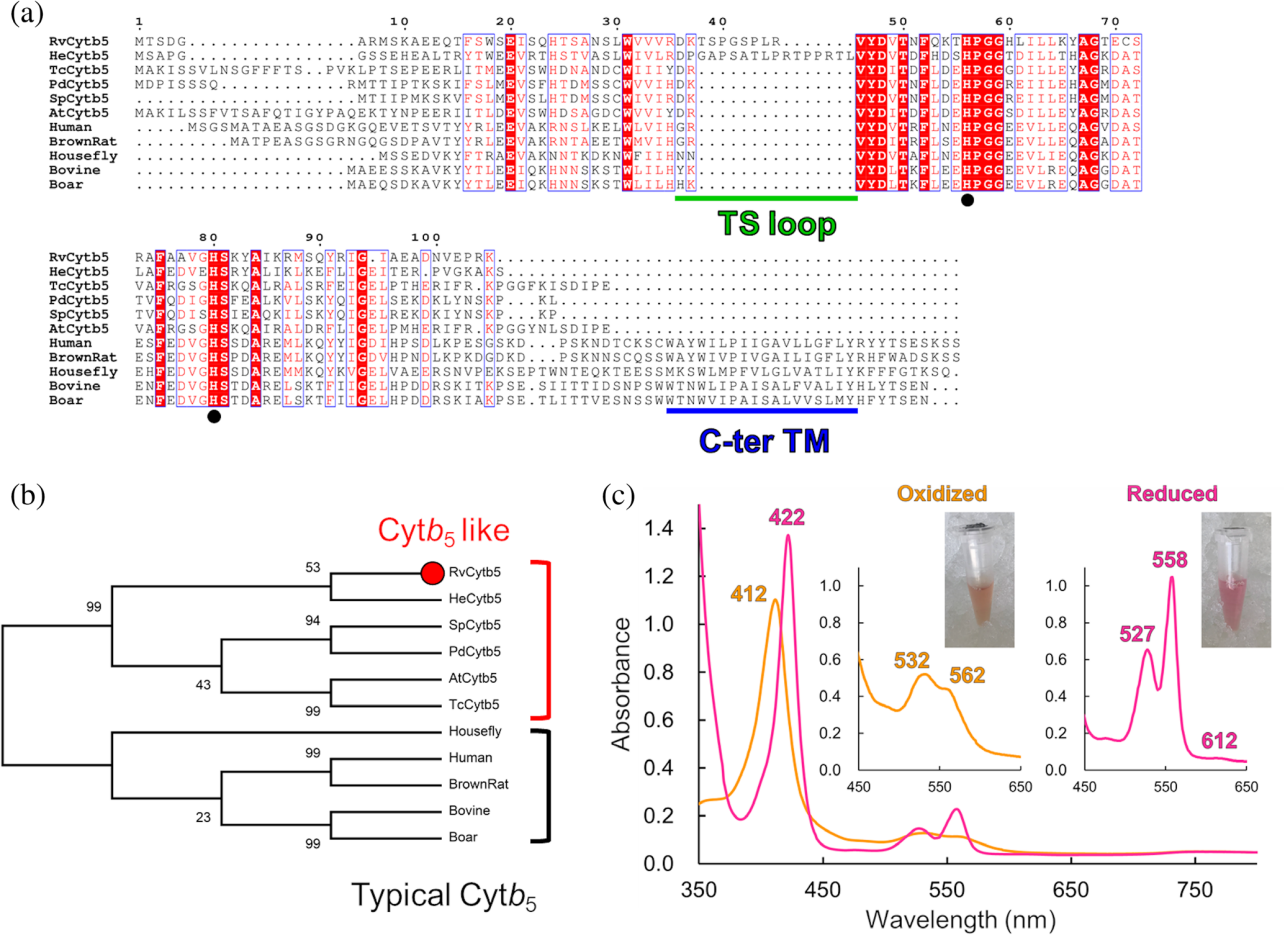
Cytochrome *b*_5_ like protein from *Ramazzottius varieornatus* (*Rv*Cyt*b*_5_). (a) Amino acid sequence alignment of *Rv*Cyt*b*_5_ with similar eukaryotic Cyt*b*_5_‐like proteins and typical Cyt*b*_5_ proteins. Rv: *Ramazzottius varieornatus*; He: *Hypsibius exemplaris* (tardigrade); Tc: *Tribolium castaneum* (insect); Pd: *Pocillopora damicornis* (coral); Sp: *Stylophora pistillata* (coral); At: *Aethina tumida* (insect). Positions of ligand histidine residues are indicated by black circles. (b) Phylogenetic tree for Cyt*b*_5_ proteins estimated by a maximum likelihood method. The percentage of trees in which the associated taxa clustered together is shown next to the branches. (c) UV–visible spectra of oxidized (orange) and reduced (pink) *Rv*Cyt*b*_5_. Insets show closeup views for the spectra recorded with five times concentrated samples and sample solutions in 1.5 ml micro tubes

The amino acid sequence of *Rv*Cyt*b*_5_ shows only 30~36% identity to typical Cyt*b*_5_ sequences. In contrast, it shows slightly higher similarity (39~46% identity) to that of a protein from a tardigrade *Hypsibius exemplaris* (*He*Cyt*b*_5_) and those of some eukaryotic proteins that are annotated as Cyt*b*_5_‐like proteins but not well characterized (Figure 1a). These Cyt*b*_5_‐like proteins are phylogenetically distinct from typical Cyt*b*_5_ proteins such as mammalian Cyt*b*_5_, structures and properties of which are extensively studied (Figure 1b). Cyt*b*_5_‐like proteins including *Rv*Cyt*b*_5_ do not have a C‐terminal transmembrane helix, which anchors the proteins on microsomal or mitochondrial membranes; therefore, they are soluble proteins. Tardigrade Cyt*b*_5_‐like proteins, *Rv*Cyt*b*_5_ and *He*Cyt*b*_5_, are distinguished from others by insertion of amino acid residues which we named tardigrade specific loop (TS loop) (Figure 1a). *Rv*Cyt*b*_5_ has a shorter TS loop than *He*Cyt*b*_5_. The TS loops contain Gly, Ser, and Pro residues, which tend to form a disordered region.

UV–visible absorption spectra were measured for *Rv*Cyt*b*_5_. The protein at its ferric (Fe^3+^) form showed a peak corresponding to a Soret band at 412 nm (Figure 1c). The peak corresponding to α and β bands were at 562 (shoulder peak) and 532 nm, respectively. The ferrous (Fe^2+^) form of *Rv*Cyt*b*_5_ exhibited Soret, α, and β band peaks at 422, 558, and 527 nm, respectively. Moreover, it had a weak peak at around 612 nm. These characteristics are similar to known cytochrome *b*_5,_ ^23^ and other bis‐histidinyl (i.e., hexacoordinated) globin proteins including neuroglobin^24^ and cytoglobin.^25^ The ratio of absorption at the β band to that at the trough between the α and β band (A_558_/A_540_) was 2.4 in the ferrous form, indicating a stable hexacoordination state.^26^

**FIGURE 2:**
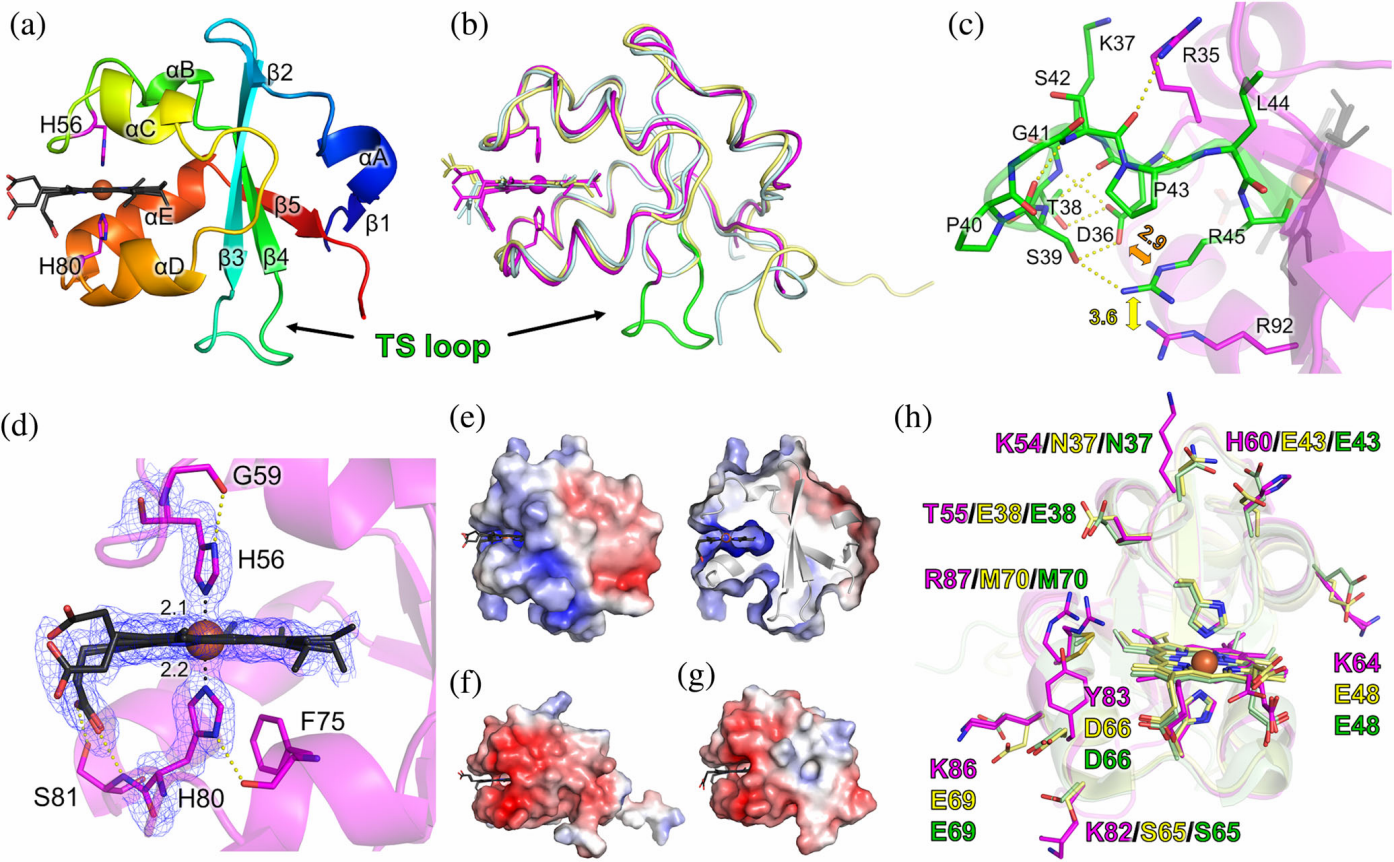
Structure of *Rv*Cyt*b*_5_. (a) Cartoon representation of the overall structure. Heme is illustrated by black sticks (carbon atoms) and a brown sphere (Fe atom). His ligands are colored by magenta. (b) Superposition of *Rv*Cyt*b*_5_ on human Cyt*b*_5_ (pale yellow: PDB code ID 3NER) and house fly Cyt*b*_5_ (pale green: PDB code ID 2IBJ). The TS loop is colored in green. (c) TS loop. Hydrogen bonds are shown by dotted yellow lines. A salt bridge is shown by orange double‐headed arrows. Arginine stacking is shown by a yellow double‐headed arrow. Distances are shown by Å unit. (d) Closeup view around the heme binding site with a sigma‐A‐weighted 2*F*_o_–*F*_c_ map (1 *σ*: blue meshes). Hydrogen bonds and coordination bonds are shown by dotted yellow and black lines, respectively. Distances are shown by Å unit (e) Electrostatic potential surface of *Rv*Cyt*b*_5_ (left) and its cross‐section view (right). The electrostatic potential is represented as a gradient from negative (red: −5 *k*_*B*_ *T*/*e*_*c*_) to positive (blue: 5 *k*_*B*_ *T*/*e*_*c*_) (f) Electrostatic potential surface of human Cyt*b*_5_. (g) Electrostatic potential surface of house fly Cyt*b*_5_. (h) Comparison of residues contributing to electrostatic potential. Magenta: *Rv*Cyt*b*_5_; yellow: human Cyt*b*_5_; green: house fly Cyt*b*_5_

The crystal structure was determined at a resolution of 1.4 Å by a single‐wavelength anomalous dispersion method using a heme iron atom (Fe‐SAD) (Table 1). The final *R*_work_ and *R*_free_ values are 0.147 and 0.171, respectively. The overall structure of *Rv*Cyt*b*_5_ is composed of five α helices and five β strands (Figure 2a), which is similar to known Cyt*b*_5_ structures such as human Cyt*b*_5_ ^27^ and house fly Cyt*b*_5_ ^28^ (Figure 2b). An obvious difference between *Rv*Cyt*b*_5_ and other typical Cyt*b*_5_ proteins is a presence of a TS loop between β3 and β4. Although the TS loop does not form any secondary structures, the residues on it form a hydrogen bond network with each other and a salt bridge between Asp36 and Arg45 (Figure 2c). The TS loop shows few interactions with surrounding residues outside the TS loop, implying that it can flexibly move independent of other parts of the protein. One interaction is a hydrogen bond formed by the carbonyl O atom of Ser42 and the side chain of Arg35. The second one is a guanidino group stacking between Arg45 and Arg92. Such kind of arginine ion pairing is reported in several proteins and is thought to stabilize protein structures.^29^

**TABLE 1:** Data collection and refinement statistics

Data collection	
X‐ray source	SPring‐8 BL44XU
Wavelength (Å)	0.9000
Space group	*P*22_1_2_1_
Unit cell *a*, *b*, *c* (Å)	34.0, 37.5, 77.5
Mosaicity (°)	0.08
Resolution range (Å)	38.8–1.40 (1.42–1.40)^a^
Total no. of reflections/no. of unique reflections	127,971 (6,055)/20,165 (969)
Completeness (%)	99.7 (100)
Redundancy	6.3 (6.2)
〈*I*/σ(*I*)〉	24.0 (3.9)
*R*_meas_ (all I+ and I−)	0.039 (0.493)
*R*_meas_ (within I+/I−)	0.038 (0.266)
CC_1/2_	0.999 (0.871)
Overall *B* factor from Wilson plot (Å^2^)	15.5

Refinement	
Resolution range (Å)	38.8–1.40 (1.436–1.400)
Completeness (%)	99.6 (99.9)
No. of reflections, working set	19,138 (1,397)
No. of reflections, test set	985 (69)
*R*_work_/*R*_free_	0.147/0.171 (0.186/0.204)
No. of non‐H atoms	1,017
Protein	791
Ion/ligand	131
Water	95
R.m.s. deviation bonds (Å), angles (°)	0.007, 1.350
Average *B* factors (Å^2^)	28.45
Protein	27.28
Ion/ligand	24.30
Water	43.95
Ramachandran favored/allowed/disallowed (%)	98.84/1.16/0
PDB code ID	7BWH

a Values in parentheses refer to the highest resolution shell.

As was expected from the spectroscopic data, hexacoordinated *b*‐type heme is observed in *Rv*Cyt*b*_5_. It is ligated by His56 and His80, which form hydrogen bonds with carbonyl O atoms of Gly59 and Phe75, respectively. The electron density map indicates that there are three different conformations of heme (Figure S1). The amino acid sequence alignment suggests that Trp18 and Tyr83 are potentially conserved residues among tardigrade Cyt*b*_5_‐like proteins (Figure 1a). Trp18 is involved in interactions between N‐ and C‐terminal regions, which are known to vary from Cyt*b*_5_ to Cyt*b*_5_.^28^ The amide N atom of Trp18 forms a hydrogen bond with the side chain carboxylate group of Glu97 near the C‐terminus (Figure S2a). Similar hydrogen bonds are observed in known Cyt*b*_5_ proteins although the positions of the residues having a carboxylate group are different from that in *Rv*Cyt*b*_5_ (Figure S2b,c). The N atom of the indole group in Trp18 interacts with the carbonyl O atom of Glu97 through a water molecule. The indole ring of Trp18 also shows a CH‐π interaction with Ile21. Tyr83 on αE indirectly interacts with amino acid residues on the same helix (Figure S2a). Moreover, this bulky Tyr residue shows a van der Waals contact with one of the methyl carbon atoms in the heme. These interactions are not observed in typical Cyt*b*_5_ proteins (Figure S2b,c). Figure 2e shows the electrostatic potential surface of *Rv*Cyt*b*_5_. In *Rv*Cyt*b*_5_, the surface surrounding the heme binding site and a cleft made by the TS loop are positively charged. Whereas, the typical Cyt*b*_5_ proteins show negatively charged surfaces around their heme binding sites (Figure 2f,g). While the surfaces of the typical Cyt*b*_5_ proteins have many residues that are negatively charged, *Rv*Cyt*b*_5_ displays positively charged residues (Figure 2h). Because the electrostatic potential surfaces of electron transfer proteins are usually optimized for their partner proteins to facilitate efficient ET complex formation, *Rv*Cyt*b*_5_ is thought to interact with proteins having negatively charged surfaces around their redox centers. That is, *Rv*Cyt*b*_5_ is probably involved in ET chains different from those related to typical Cyt*b*_5_ proteins. Considering that *Rv*Cyt*b*_5_ is a tardigrade‐specific protein, it might be involved in biological processes unique to tardigrades. Identification of the partner protein(s) for *Rv*Cyt*b*_5_ is under way.

## 3 MATERIALS AND METHODS

### 3.1 *Sequence alignment*

Homology analyses were performed by BLAST.^30^ Sequence alignment was performed by Clustal Omega^31^ for structurally characterized Cyt*b*_5_ proteins and Cyt*b*_5_‐like proteins showing the top five scores in the BLAST analysis. The alignment figure was generated by ESpript.^32^ Phylogenetic analysis by a maximum likelihood method was conducted in MEGA X.^33^

### 3.2 *Protein expression and purification*

The GenBank accession ID of the structural gene for *Rv*Cyt*b*_5_ from *R. varieornatus* is GAV03092.1. A synthesized and codon optimized DNA coding *Rv*Cyt*b*_5_(10–102) was purchased from GenScript. The gene was cloned into a pET28a vector. A 6× His tag followed by a tobacco etch virus (TEV) protease site (ENLYFQS) was attached at the N‐terminus of *Rv*Cyt*b*_5_(10–102) for purification. Its complete sequence is shown in Appendix S1 of Supporting Information. The protein was expressed in *Escherichia coli* BL21 Star(DE3) (Invitrogen, Waltham, MA). At culture optical density of ~0.6, 0.5 mM isopropyl β‐D‐1 thiogalactopyranoside along with 0.5 mM aminolevulinic acid was added to induce expression. After 18 hr at 18°C, the bacterial pellet was collected and then sonicated in a buffer containing 20 mM Tris–HCl pH 8, 300 mM NaCl, benzonase (Merck Millipore, Burlington, MA), and a complete Protease Inhibitor Cocktail tablet (Roche, Basel, Basel‐Stadt, Switzerland). The resulting solution was centrifuged and supernatant was purified using a HiTrap TALON column (GE healthcare, Chicago, IL). A buffer used to equilibrate the column and wash the sample consisted of 20 mM Tris–HCl pH 8 and 5 mM imidazole. A buffer used for elution consisted of 20 mM Tris–HCl pH 8 and 200 mM imidazole. The sample was incubated with TEV protease and imidazole was removed through dialysis against 20 mM Tris–HCl pH 8 overnight at room temperature. The sample was then loaded on a HisTrap column (GE healthcare) equilibrated by 20 mM Tris–HCl pH 8 and 40 mM imidazole. The flow‐through fraction was dialyzed against 50 mM Phosphate buffer pH 6.0 and purified by a HiTrap SP column (GE healthcare). The fractions containing *Rv*Cyt*b*_5_ were collected and further purified using a Hiload 16/60 Superdex 75 gel filtration column (GE healthcare). As for gel filtration, 20 mM Tris–HCl buffer pH 8 was used.

### 3.3 *Crystallization*

Crystallization was performed by the sitting drop vapor‐diffusion method. A crystallization machine mosquito (TTP LabTech, Melbourn, Hertfordshire, UK) was used to prepare drops on 96‐well VIOLAMO plates (AS ONE, Osaka, Osaka, Japan). The reservoir solution was 60 μl, and 0.1 μl protein solution was mixed with 0.1 μl reservoir solution. After 4 months, a crystal appeared under the condition of 26 mg/ml *Rv*Cyt*b*_5_, 22% (w/v) polyethylene glycol 4000, and 50 mM 4‐(2‐hydroxyethyl)‐1‐piperazineethanesulfonic acid (HEPES) buffer pH 7.5 at 20°C. Before the crystal was frozen by liquid nitrogen, it was soaked in the crystallization solution supplemented by 15% v/v ethylene glycol.

### 3.4 *X‐ray data collection, processing, structure solution, and refinement*

X‐ray diffraction experiment was performed on the BL44XU beamline of SPring‐8, Hyogo, Japan. Diffraction images were collected at 100 K using an EIGER X 16 M detector (Dectris, Philadelphia, PA). A 0.8 mm Al attenuator was used to weaken X‐ray. The crystal‐to‐detector distance was 160 mm. The exposure time per frame and the oscillation angle were 0.1 s and 0.1°, respectively. The dataset was processed using XDS^34^ and scaled by Aimless.^35^ Phase determination and initial model building was performed by CRANK2.^36^ Manual model building was performed using Coot.^37^ The program refmac5 in the ccp4 suite^38^ and the program phenix.refine^39^ were used for structural refinement. Anisotropic parameters were introduced because of its high resolution. The stereochemical quality of the final model was checked by Molprobity.^40^ Data collection and refinement statistics are summarized in Table 1. The coordinate and structure factor files are deposited at the Protein Data Bank (PDB code ID: 7BWH). Raw data is available at Integrated Resource for Reproducibility in Macromolecular Crystallography (https://proteindiffraction.org/).

### 3.5 *UV–visible absorption spectroscopy*

The samples were in a 1 cm quartz cell. UV–visible absorption spectra of oxidized *Rv*Cyt*b*_5_ were recorded in 20 mM HEPES pH 7.1 with spectramax M2 and softmax pro 5.4 software (Molecular Devices, San Jose, CA) at room temperature. UV–vis spectrum of reduced *Rv*Cyt*b*_5_ in 20 mM HEPES pH 7.1 was recorded by adding 5 mM sodium dithionite at room temperature.

## AUTHOR CONTRIBUTIONS

**Yohta Fukuda:** Conceptualization; data curation; formal analysis; funding acquisition; investigation; visualization; writing‐original draft. **JeeEun Kim:** Data curation; formal analysis; investigation; writing‐review and editing.

## CONFLICT OF INTEREST

The authors declare no competing financial interests.

## Supporting information


**Appendix S1** Supporting Information
