# Supplementary material for: Structure of cytochrome b 5 unique to tardigrades
Source: Protein Sci. 2020 Jul 24;29(8):1829–35. doi: 10.1002/pro.3896 (PMC7380671; doi:10.1002/pro.3896)
Supplement: Supplementary file 1 — Appendix S1 Supporting Information [file PRO-29-1829-s001.docx]

**Supporting Information**

**Appendix S1. Sequence of *Rv*Cyt*b*_5_**

**Original sequence of *Rv*Cyt*b*_5_ (GAV03092.1 hypothetical protein RvY_13572):**

MTSDGARMSKAEEQTFSWSEISQHTSANSLWVVVRDKTSPGSPLRVYDVTNFQKTHPGGHLILLKYAGTECSRAFAAVGHSKYAIKRMSQYRIGIAEADNVEPRK

**Amino acid sequence of expression construct:**

MGSSHHHHHHHSSENLYFQSLRKDKAEEQTFSWSEISQHTSANSLWVVVRDKTSPGSPLRVYDVTNFQKTHPGGHLILLKYAGTECSRAFAAVGHSKYAIKRMSQYRIGIAEADNVE

**DNA sequence of expression construct:**

ATGGGCAGCAGCCATCATCATCATCATCACAGCAGCGAAAACCTGTATTTTCAGTCCCTGCGTAAAGATAAGGCGGAGGAACAGACCTTCAGCTGGAGCGAGATCAGCCAACACACCAGCGCGAACAGCCTGTGGGTGGTTGTGCGTGACAAAACCAGCCCGGGTAGCCCGCTGCGTGTGTACGATGTTACCAACTTCCAGAAGACCCACCCGGGTGGCCACCTGATCCTGCTGAAATATGCGGGTACCGAATGCAGCCGTGCGTTTGCGGCGGTGGGTCACAGCAAGTACGCGATTAAACGTATGAGCCAATATCGTATCGGCATTGCGGAGGCGGACAACGTGGAGTAA

Green letters: sequence of RvCyt*b*_5_(10-102)

Blue letters: TEV protease site

LRKD: extra residues to enhance expression

Underline: His tag

TAA: stop codon

Supplementary Figure


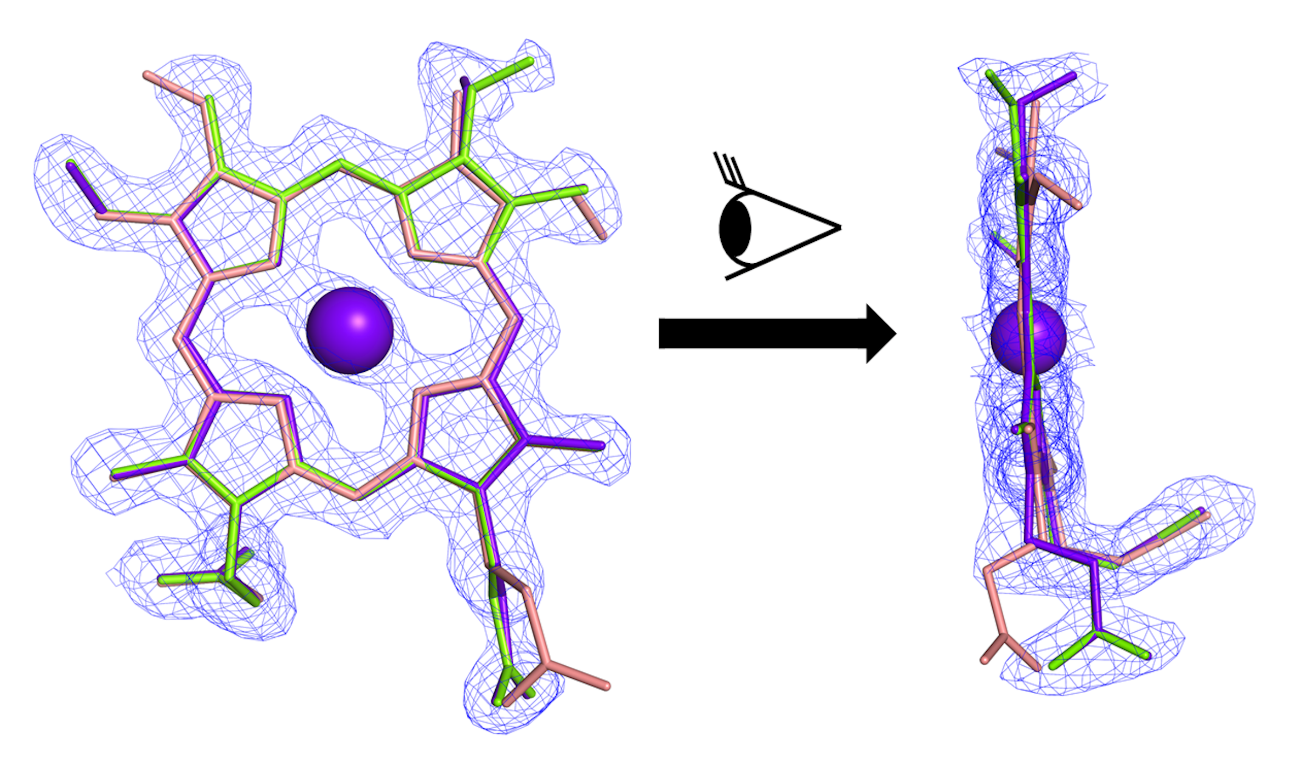


**Figure S1. Heme conformations in *Rv*Cyt*b*_5_.** Sigma-A-weighted 2*F*_o_-*F*_c_ map at 1 sigma is illustrated by blue meshe. Three different conformations are colored by pink, green, and purple, respectively.


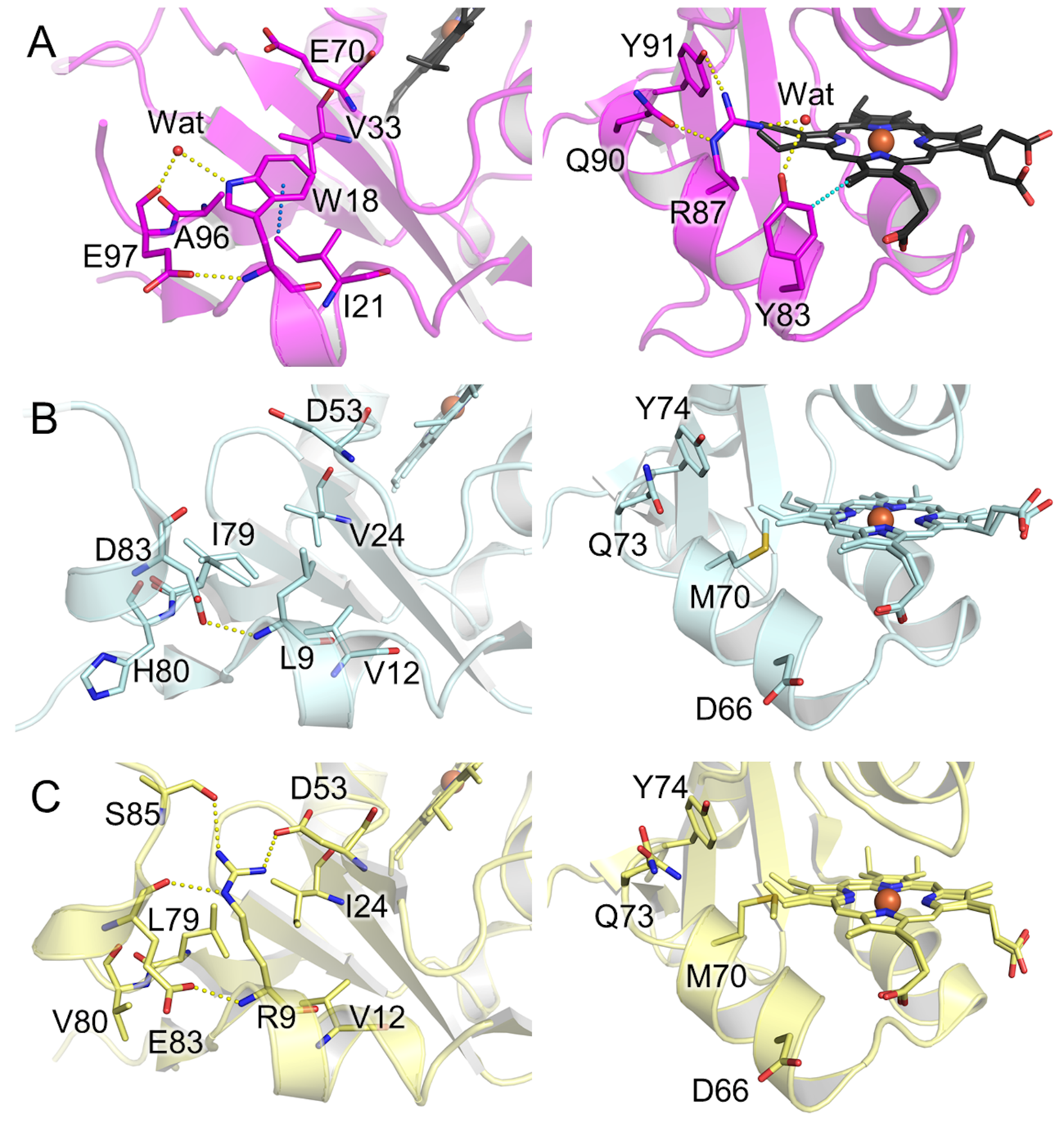


**Figure S2. Possible tardigrade-specific residues and their interactions with surrounding residues.** (A) Trp18 and Tyr83 in *Rv*Cyt*b*_5_ shown with observed interactions. (B) Corresponding region to (A) in human Cyt*b*_5_. (C) Corresponding region to (A) in house fly Cyt*b*_5_. Hydrogen bonds, van der Waals contact, and CH-π interaction are illustrated by dotted yellow, cyan, and blue lines, respectively.
